# Elevated A-to-I RNA editing in COVID-19 infected individuals

**DOI:** 10.1093/nargab/lqad092

**Published:** 2023-10-18

**Authors:** Rona Merdler-Rabinowicz, David Gorelik, Jiwoon Park, Cem Meydan, Jonathan Foox, Miriam Karmon, Hillel S Roth, Roni Cohen-Fultheim, Galit Shohat-ophir, Eli Eisenberg, Eytan Ruppin, Christopher E Mason, Erez Y Levanon

**Affiliations:** Mina and Everard Goodman Faculty of Life Sciences, Bar-Ilan University, Ramat Gan, Israel; Cancer Data Science Lab, Center for Cancer Research, National Cancer Institute, National Institutes of Health, Bethesda, MD, USA; The Institute of Nanotechnology and Advanced Materials, Bar‐Ilan University, Ramat Gan, Israel; Mina and Everard Goodman Faculty of Life Sciences, Bar-Ilan University, Ramat Gan, Israel; The Institute of Nanotechnology and Advanced Materials, Bar‐Ilan University, Ramat Gan, Israel; Department of Physiology, Biophysics and Systems Biology, Weill Cornell Medicine, New York, NY, USA; Laboratory of Virology and Infectious Disease, The Rockefeller University, New York, NY, USA; Department of Physiology, Biophysics and Systems Biology, Weill Cornell Medicine, New York, NY, USA; Department of Physiology, Biophysics and Systems Biology, Weill Cornell Medicine, New York, NY, USA; Mina and Everard Goodman Faculty of Life Sciences, Bar-Ilan University, Ramat Gan, Israel; The Institute of Nanotechnology and Advanced Materials, Bar‐Ilan University, Ramat Gan, Israel; Mina and Everard Goodman Faculty of Life Sciences, Bar-Ilan University, Ramat Gan, Israel; The Institute of Nanotechnology and Advanced Materials, Bar‐Ilan University, Ramat Gan, Israel; Mina and Everard Goodman Faculty of Life Sciences, Bar-Ilan University, Ramat Gan, Israel; The Institute of Nanotechnology and Advanced Materials, Bar‐Ilan University, Ramat Gan, Israel; Mina and Everard Goodman Faculty of Life Sciences, Bar-Ilan University, Ramat Gan, Israel; Leslie and Susan Gonda Multidisciplinary Brain Research Center and The Nanotechnology Institute, Bar-Ilan University, Ramat Gan, Israel; Raymond and Beverly Sackler School of Physics and Astronomy, Tel-Aviv University, Tel Aviv, Israel; Cancer Data Science Lab, Center for Cancer Research, National Cancer Institute, National Institutes of Health, Bethesda, MD, USA; Department of Physiology, Biophysics and Systems Biology, Weill Cornell Medicine, New York, NY, USA; The HRH Prince Alwaleed Bin Talal Bin Abdulaziz Alsaud Institute for Computational Biomedicine, Weill Cornell Medicine, New York, NY, USA; Caryl and Israel Englander Institute for Precision Medicine, Weill Cornell Medicine, New York, NY, USA; The WorldQuant Initiative for Quantitative Prediction, Weill Cornell Medicine, New York, NY, USA; Mina and Everard Goodman Faculty of Life Sciences, Bar-Ilan University, Ramat Gan, Israel; The Institute of Nanotechnology and Advanced Materials, Bar‐Ilan University, Ramat Gan, Israel

## Abstract

Given the current status of coronavirus disease 2019 (COVID-19) as a global pandemic, it is of high priority to gain a deeper understanding of the disease's development and how the virus impacts its host. Adenosine (A)-to-Inosine (I) RNA editing is a post-transcriptional modification, catalyzed by the ADAR family of enzymes, that can be considered part of the inherent cellular defense mechanism as it affects the innate immune response in a complex manner. It was previously reported that various viruses could interact with the host's ADAR enzymes, resulting in epigenetic changes both to the virus and the host. Here, we analyze RNA-seq of nasopharyngeal swab specimens as well as whole-blood samples of COVID-19 infected individuals and show a significant elevation in the global RNA editing activity in COVID-19 compared to healthy controls. We also detect specific coding sites that exhibit higher editing activity. We further show that the increment in editing activity during the disease is temporary and returns to baseline shortly after the symptomatic period. These significant epigenetic changes may contribute to the immune system response and affect adverse outcomes seen in post-viral cases.

## Introduction

Coronavirus disease 2019 (COVID-19) is a contagious disease caused by an infection with the severe acute respiratory syndrome coronavirus 2 (SARS-CoV-2) virus strain. The disease primarily involves the respiratory system, causing mild to moderate symptoms in most cases. However, deterioration towards a multi-systemic disease may also occur, resulting in multi-organ failure and even death in severe cases (1). The severity of the disease, including morbidity and mortality rates, is directly linked to the extent of the host immune response, which is called the "cytokine storm," characterized by elevated serum levels of pro-inflammatory cytokines (2). As COVID-19 has become an uncontrolled emerged pandemic worldwide, it is of great interest to thoroughly understand the disease's pathogenesis, particularly the components of the immune system that are associated with a severe state.

Typically, the immune system's response to viruses is preceded by an interferon (IFN)-mediated response, which plays a critical role in regulating immune activity to enhance host protection and minimize collateral damage (3,4). The role of IFN in COVID-19 is being investigated (5,6).

RNA editing is a mechanism that enables RNA modifications in the transcriptome. Several types of RNA editing have been recognized so far. In humans, adenosine-to-inosine (A-to-I) is the most abundant RNA editing process, carried by the Adenosine Deaminase Acting on RNA (ADAR) family of enzymes, particularly by ADAR1, which is responsible for the majority of the editing activity (7). Because most cellular machineries interpret inosine as a guanosine (G), this deamination can potentially result in altered splice consensus elements (8), microRNA seeds (9,10), protein binding sites (11) and coding sequences (12). *ADAR1* mediates editing on endogenous double-stranded RNA (dsRNA) structures, thus protecting the cell against activation of the dsRNA sensor Melanoma Differentiation-Associated Protein 5 (MDA5), a protein that binds long dsRNA structures, that usually characterize viral structures, and in turn activates the antiviral cellular immune system including a strong interferon response, in order to induce host-against virus reaction. In other words, proper *ADAR1* editing is vital for preventing immune system activation against self (13–15). *ADAR1* knockout cells exhibit early cell death (16,17), and specific mutations in *ADAR1* lead to Aicardi–Goutières syndrome - an autoinflammatory disorder characterized by dysregulation of IFN-I activity (18).

The ideal target for *ADAR* enzymes is a long dsRNA duplex. In humans, >99% of the millions of recognizable editing sites are located in *Alu*-repeats (19–22), which are short interspersed DNA elements (SINEs) composed of repetitive sequences. *Alu* repeats constitute about 10% of the human genome, typically located in introns of genome-rich areas (23). Due to its repetitive nature, *Alu*-elements are often transcribed aside with a similar inversed copy, and these often form a dsRNA structure.

Two main different isoforms of *ADAR1* were identified: ADARp110, which is confined to the nucleus, and ADARp150, which shuttles to the cytoplasm and is induced by IFN (24). As most viruses replicate in the cytoplasm, the cytoplasmic ADARp150 is likely responsible for viral genome editing. The observation that IFNs induces ADARp150 expression (25) further supports the hypothesis that this isoform plays a role in the host defense mechanism against viruses.

All viruses that have dsRNA structures at any stage of their life cycle can potentially undergo RNA editing events mediated by ADAR enzymes, as was reported for several viruses (26,27), and was investigated regarding SARS-CoV-2 (28,29). It is reasonable to assume that this viral-induced overexpression of host ADAR enzymes will lead to increased RNA editing of the human transcriptome as well (30), and result in uncontrolled epigenetic modifications.

In the current work we analyze A-to-I RNA editing patterns in COVID-19, to further elucidate the effect of SARS-CoV-2 on the host. Using multiple computational approaches on several RNA-seq datasets, we show that the editing levels are dramatically increased during the disease.

## Materials and methods

### Datasets

The primary nasopharyngeal-swabs dataset was downloaded from the database of Genotypes and Phenotypes dbGAP (accession #38851 and ID phs002258.v1.p1) (31). This dataset contained total RNA-seq files of nasopharyngeal swab specimens and oropharyngeal swab lysates extracted from 221 cases of COVID-19, 92 cases of other-viral respiratory infections (several common cold coronavirus strains and influenza virus, as detected by BioFire PCR panel) and 419 controls. As outlined in the initial research publication, the COVID-19 samples were quantified by RNA-seq (log_10_ SARS-CoV-2 % of reads) and qRT-PCR (Ct values) to create a three-tier range of viral load (low, medium, high).

In total, 2 COVID-19, 1 other-viral respiratory infection, and 14 control cases failed to run our pipeline, thus the analysis was performed on 219 COVID-19 cases, 91 other-viral respiratory infection cases, and 405 controls.

Another smaller nasopharyngeal-swabs dataset that was downloaded from the database of Gene Expression Omnibus (GEO) (accession # GSE166530) (32) contained 36 COVID-19 positive patients and 5 COVID-19 negative controls.

Three whole-blood datasets were downloaded from the GEO database. The first dataset (accession # GSE167000) (33–35) contained 65 samples of hospitalized COVID-19 positive subjects and 30 COVID-19 negative subjects as controls. The second dataset (accession # GSE171110) (36) had 44 samples of COVID-19 patients with severe disease (all met the criteria of the French COVID cohort (37)) and 10 healthy controls. A third dataset (accession # GSE157103) (38) contained leukocytes samples of 100 COVID-19 patients and 26 non-COVID-19 patients.

Last, an RNA-seq dataset of long COVID-19 was downloaded from the GEO (accession # GSE166190) (39). It contained blood samples of 21 infected individuals and 4 close-contact non-infected controls over time, up to eight weeks post symptoms. Sampling was done in 5-time intervals according to the calculated days post symptoms: period 1 (0–5 days), period 2 (6–14 days), period 3 (15–22 days), period 4 (23–35 days) and period 5 (36–81 days).

### Pre-processing, alignment, and filtering of the data

The Prinseq-lite program (40) was used to remove PCR duplicate reads. Sequence reads were aligned to the hg38 reference genome, using STAR (41), taking only uniquely mapped reads (outFilterMultimapNmax = 1) and using the suggested parameters. RNA editing detection tools were used, as described here. If more than one sample was sequenced from the same individual, the average value of the samples was used for each test.

### Gene expression

The Salmon software tool (42) was used to quantify transcripts’ expression levels. *ADAR1, ADAR2*, and *ADAR3* expressions were evaluated. Statistical analysis was performed using R.

### Interferon signature

To explore the status of IFN signaling, we assessed expression profiles of interferon-stimulated genes (ISG) and derived a score using a 38-gene signature. ISG scores (mean absolute deviation modified Z-score) were calculated as previously described (43,44). We also investigated expression levels of IFN lambda and IFN beta genes. We used the output from the Salmon program (42) as input for the ISG pipeline. Differences between the groups were statistically analyzed using R.

### Global editing index

As the vast majority of RNA editing activity in primates takes place in *Alu* repetitive elements, we evaluated the relative editing levels of *Alu* elements in the tested samples. In theory, the collection of REDiportal (45) RNA editing-prone sites could be utilized for this objective, as the majority of sites are located in *Alu* elements. However, the detection of editing in *Alu* regions may be subject to bias due to the typically low sequencing coverage in these areas. Thus, we used the *Alu* editing index (AEI), a previously published and validated method by our group, as the most reliable approach for detecting editing levels (46). This index is calculated by measuring the average editing of *Alu* adenosines, weighted by their expression levels. Since this index is calculated from the averages of millions of adenosines, it is rather robust to statistical noise.

Differences in the A-to-G editing index were statistically analyzed using R.

### Editing in known coding sites

We analyzed the RNA editing levels in a list of specific coding sites that our group recently published to form high editing indices (12). We used the RedIToolsKnown tool, which is part of the REDItools package (47). For each coding site, we analyzed only sites covered by at least 20 mapped reads per sample in at least 10 mapped samples per group. Differences between the groups were statistically analyzed using R.

### Statistical analysis and graphs

Graphs and statistical analyses were obtained using R (R Foundation for Statistical Computing (https://www.R-project.org/)). The Wilcoxon Test or the *t*-test followed by FDR multiple-testing correction were used to evaluate statistical changes between groups. Correlations were evaluated using the Pearson correlation test.

### Source code

The source code used to produce the results and analyses presented in this manuscript are available on a GitHub repository at: https://github.com/zbidav/COVID19-rawData.git

## Results

### Enhanced global RNA editing in COVID-19 patients

**Figure 1.**
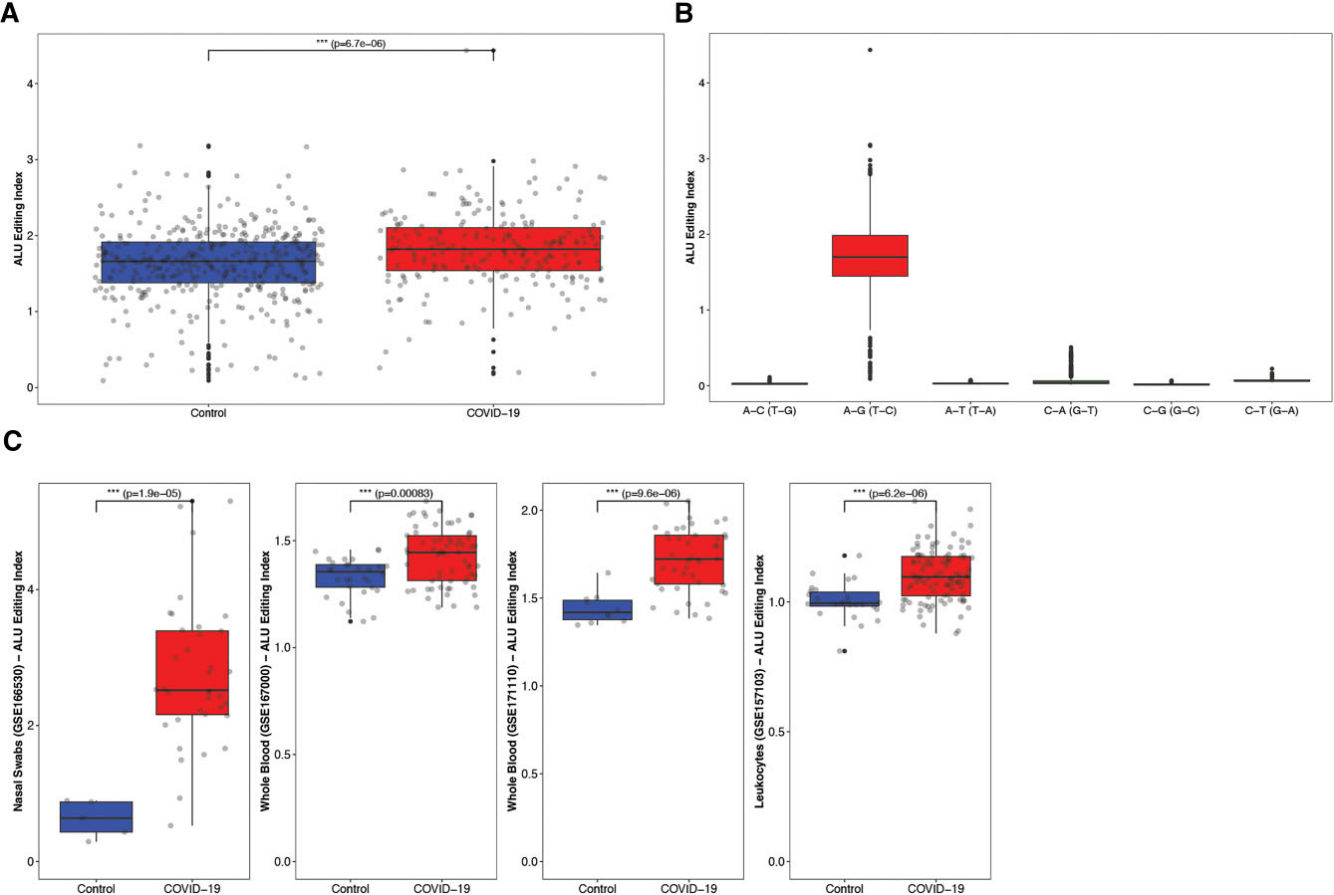
Global A-to-I editing in *Alu* elements. (**A**) The *Alu* editing index (AEI) in nasal swabs of COVID-19 patients is significantly elevated compared to controls. (**B**) The high index of A-to-G mismatch, compared to any other possible mismatch, is indicative to the cleanness of the A-to-I editing signal. (**C**) The *Alu* editing index (AEI) is significantly elevated in COVID-19 compared to controls, in 4 different datasets of whole blood, leukocytes and nasal-swabs.

We used the RNA sequencing dataset of nasal-swabs that was previously published by Butler *et al.* (31) to compare the RNA-editing levels of 219 COVID-19 patients with those of 91 other-viral respiratory cases and 405 controls . We calculated the *Alu* editing index (AEI) for each individual, which measures the global rate of editing in Alu repeats (48). The COVID-19 group had significantly higher AEI than the controls (Wilcoxson; *P*-value = 6.7e-06) (Figure 1A). Overall, the accuracy of the editing detection in *Alu* regions was extremely high as the A-to-G index, indicative of A-to-I editing, was way higher than for any other possible mismatches (Figure 1B).

Similar results were obtained from another smaller nasal-swabs dataset of 36 COVID-19 patients and 5 controls, and three whole-blood datasets that were also extracted from COVID-19 patients and controls. The COVID-19 group had significantly elevated AEI compared to the matched control group in all cases (Figure 1C). In general, the differences between the groups were much more evident in the nasal swab datasets, probably since the upper respiratory epithelium is directly attacked and affected by the virus.

There was no correlation between AEI levels and sex or age, both in the COVID-19 group and the control group, indicating that the editing levels are not affected by these factors (Supplementary Figure S1).

Unlike the majority of *Alu* elements that are located in introns thus are removed during the transcription process, *Alu* elements that are located in 3'UTR regions are shuttled to the cytoplasm where they can be edited by the ADAR1 p150 isoform - an isoform that is highly activated by IFN. Therefore, to further strengthen the correlation between COVID-19 disease and increased RNA editing activity, we focused on specific 3'UTR regions that are rich with *Alu* elements, which form long and stable dsRNA structures in the cytoplasm and are predicted to induce a strong editing activity.

**Figure 2.**
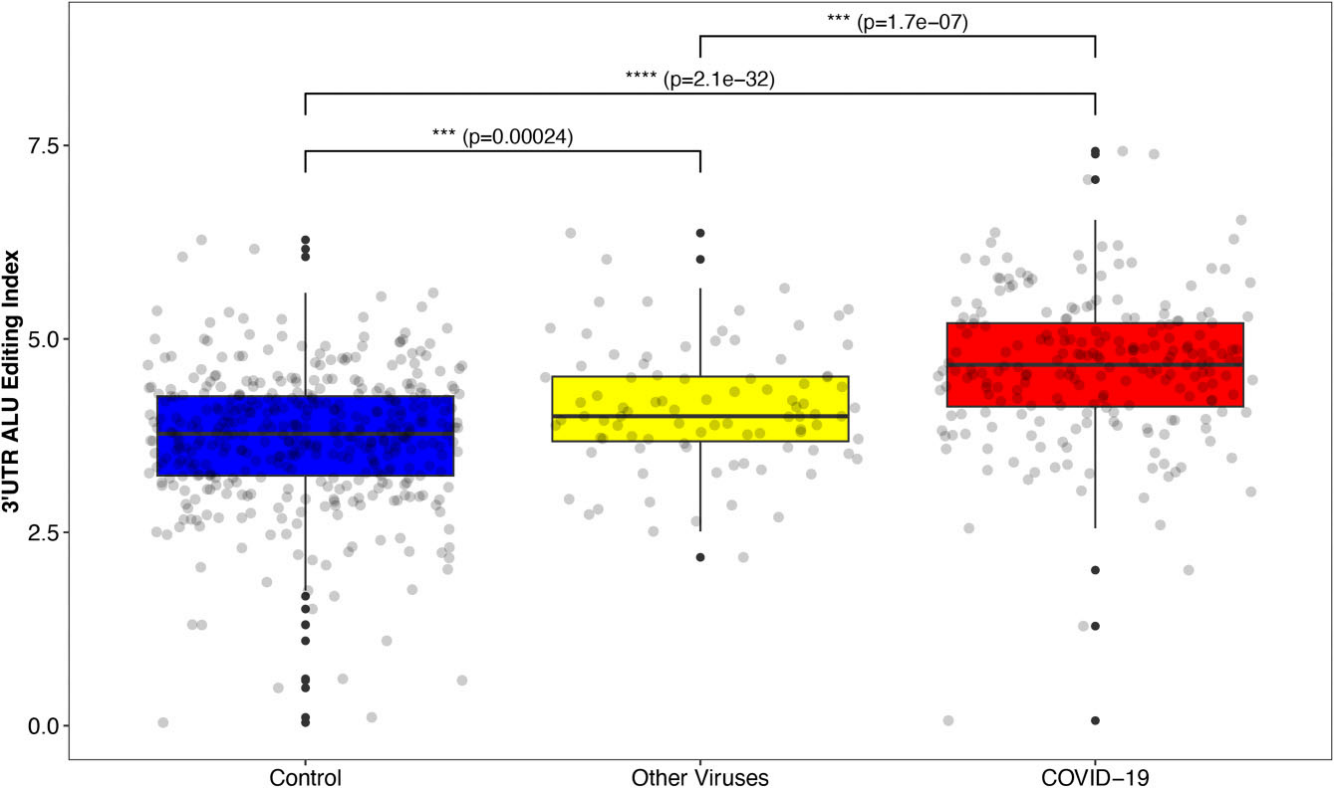
A-to-I editing in *Alu* elements that are located in 3'UTR regions (3'UTR-AEI). *Alu* elements that are located in 3'UTR regions are predicted to form long dsRNA structures that induce strong editing activity. A significant elevation in 3'UTR-AEI is shown in the COVID-19 group compared both to the other respiratory-viruses group and to controls.

Indeed, comparing the editing index calculated for this subset of repetitive elements, the differences between the groups grew even higher. The UTR-Alu editing index in the COVID-19 group was much higher than the global index (COVID-19 group; global AEI: mean = 1.82, median = 1.82. 3'UTR-AEI: mean = 4.64, median = 4.66), and was significantly higher compared with controls (Wilcoxson; *P*-value = 2.1e-32) (Figure 2).

We further compared the COVID-19 group to the other-viral respiratory group, that consists of several common cold coronavirus strains and influenza virus. We found a significant 3'UTR-AEI elevation among the COVID-19 group compared to the other-viral respiratory group (Wilcoxson; *P*-value = 1.7e-07). The latter also demonstrated a significantly enhanced 3'UTR-AEI compared to controls, but to a lesser extent (Wilcoxson; *P*-value = 2.4e-04) (Figure 2, Supplementary Figure S2). These findings further emphasize the immense effect of COVID-19 infection on the host editing activity, even in comparison to other quite similar viruses.

**Figure 3.**
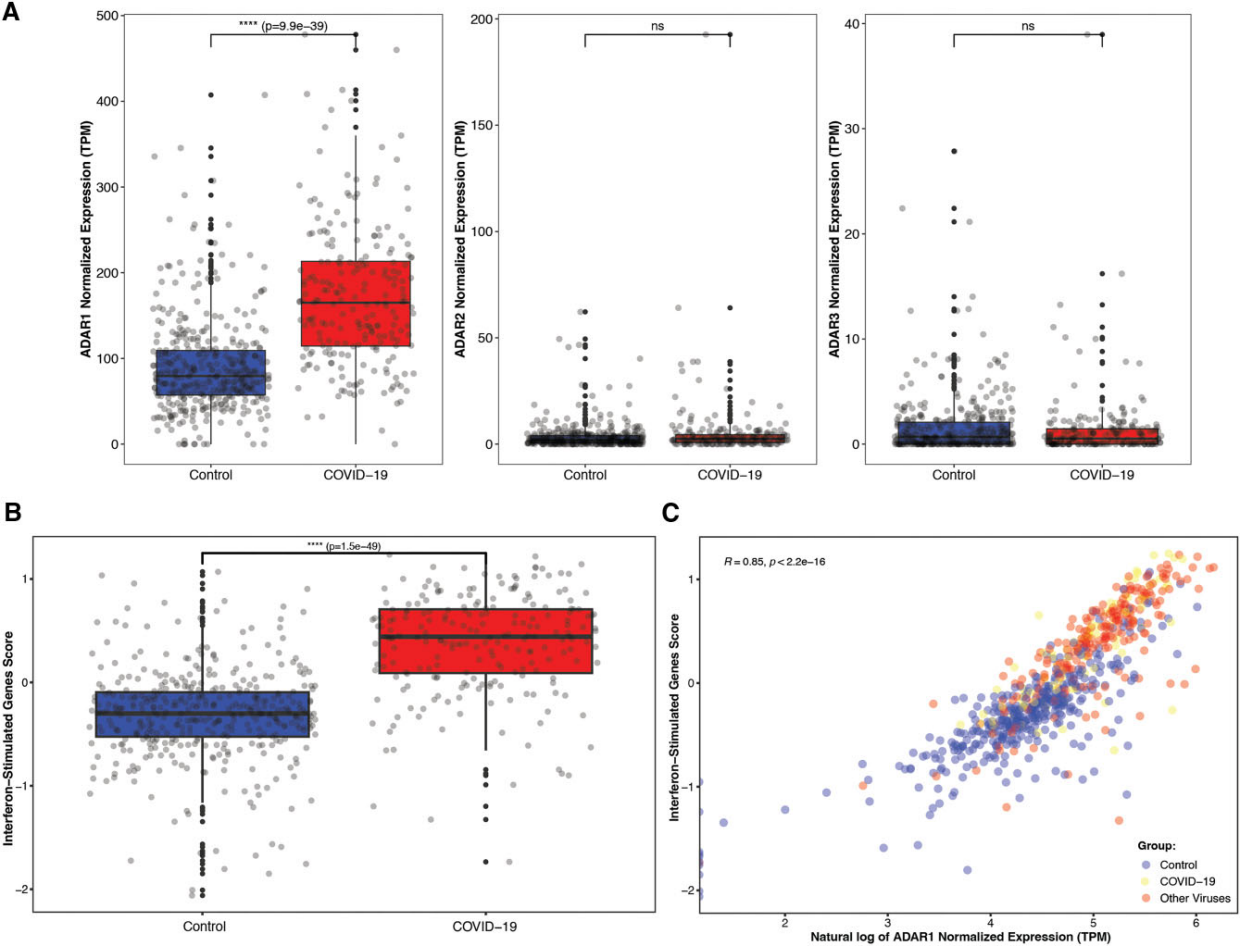
ADAR enzymes and other interferon-stimulating genes expression levels and correlations. (**A**) Expression levels of ADAR1, ADAR2 and ADAR3 in the COVID-19 group compared to controls. (**B**) Calculated interferon-stimulated genes (ISG38) score, as derived from a 38-genes signature, in the COVID-19 group compared to controls. The result is significantly higher in the COVID-19 group. (**C**) Correlation between ADAR1 expression levels and ISG38 score, for the COVID-19 group (red dots), the other respiratory-viruses group (yellow dots) and the controls (blue dots).

To determine whether ADAR1, which is stimulated by IFN, is the enzyme responsible for the increased editing levels, we examined its expression levels. As expected, its expression was significantly higher in the COVID-19 group compared to controls (Wilcoxson; COVID-19: 172.51 ± 83.38 TPM, controls: 90.44 ± 55.96 TPM. *P*-value = 9.94e-39). The expression levels of the other ADAR family members—ADAR2 (*ADARB1*) and ADAR3 (*ADARB2*)—which are not stimulated by IFN—were much lower compared to ADAR1 and demonstrated a more complex pattern of expression (Figure 3A, Supplementary Figure S3).

Last, to further demonstrate the link between ADAR1 and IFN, we evaluated the IFN expression profile by calculating the interferon-stimulated genes (ISG) score derived from a 38-genes signature (43,44). As expected, the score was significantly higher in the COVID-19 group compared to the control group (Wilcoxson; COVID-19: 0.37 ± 0.5, controls: –0.32 ± 0.46, *P*-value = 1.5e-49) (Figure 3B) as ADAR1 expression levels were highly correlated with ISG38 score (Spearman; *R* = 0.85, *P* < 2.2e-16) (Figure 3C). INF lambda and INF beta levels were also investigated and were found to be elevated among the COVID-19 group (Supplementary Figure S4). Since the only difference between the two main *ADAR1* isoforms, ADAR1 p150 and ADAR1 p110, is the existence or absence of the first exon, it is difficult to determine the expression levels of each isoform based on short-reads RNA-seq data. However, the fact that IFN induces ADAR1-p150 only, and our findings that mRNA cytoplasmatic molecules are highly edited in COVID-19, imply that ADAR1-p150 is indeed the main isoform that is responsible for the wide extent of editing activity seen during COVID-19 infection.

**Figure 4.**
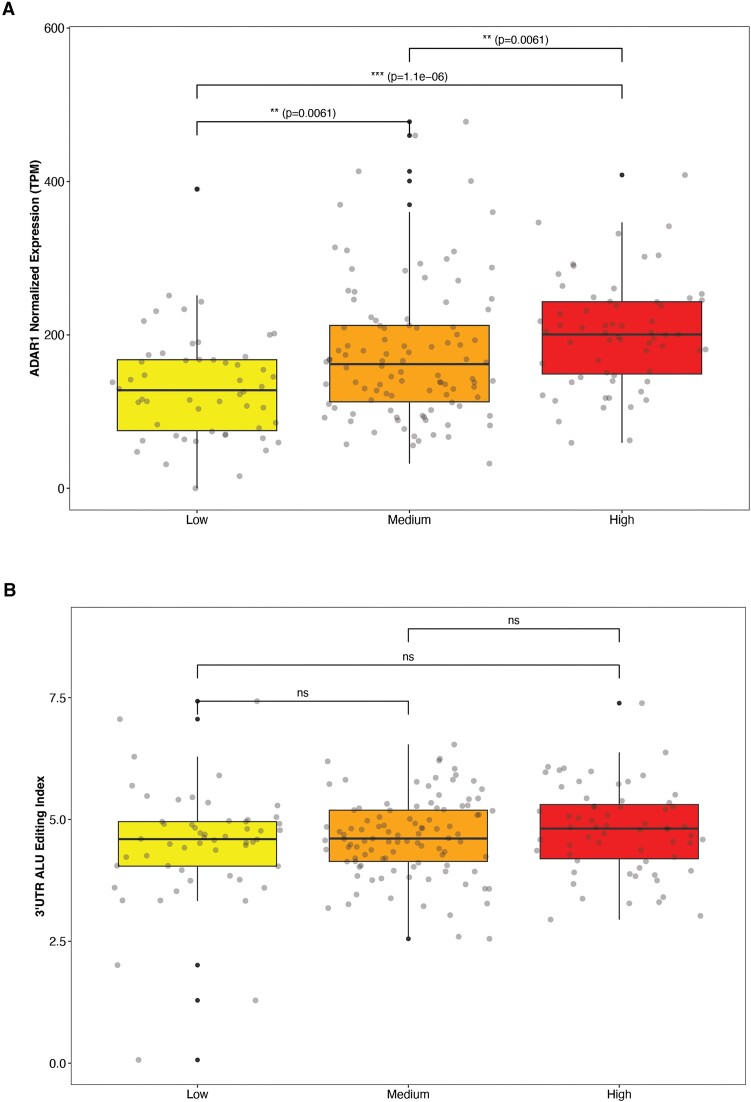
Genes expression and editing index in the context of SARS-CoV-2 viral load. (**A**) The expression levels of ADAR1 as assessed in three distinct sub-groups of COVID-19 based on their viral load. (**B**) The Editing index of *Alu* elements located in 3'UTR regions (3'UTR-AEI) as assessed in three distinct sub-groups of COVID-19 based on their viral load.

**Figure 5.**
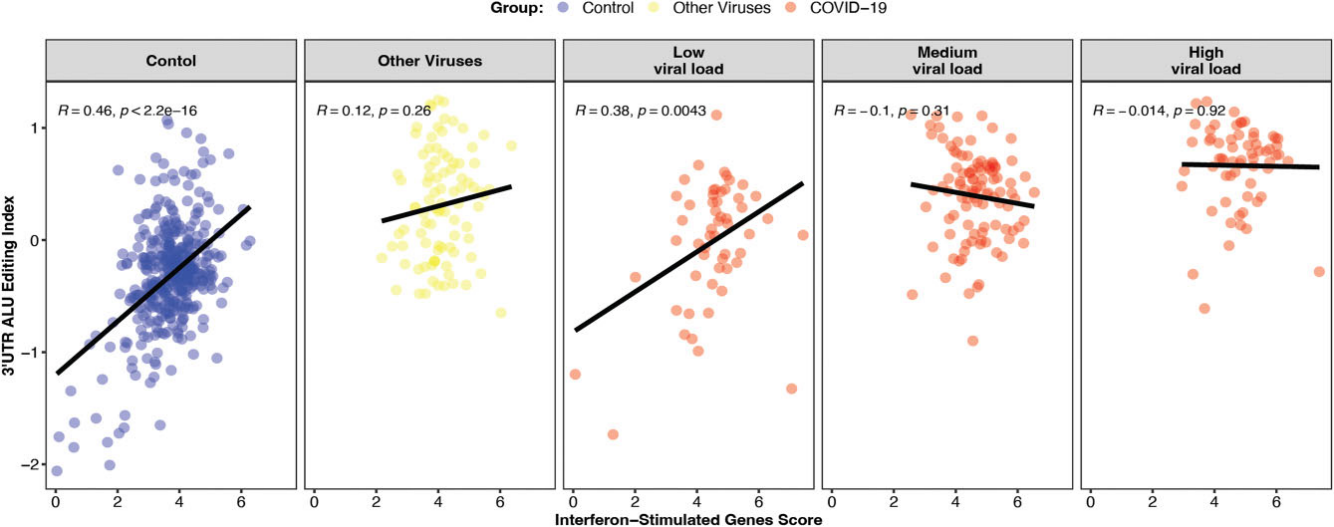
Correlation between ISG-38 score and 3'UTR-AEI. The Correlation between ISG-38 score and 3'UTR-AEI, shown for three distinct sub-groups of COVID-19 based on their viral load and for the controls.

To study the impact of SARS-CoV-2 viral load on host editing, we employed the categorization established in the original publication (31), utilizing RNA-seq and qRT-PCR analyses of COVID-19 samples to sort them into three viral load subgroups (low, medium, high). We found that while ADAR1 expression levels increase with higher viral load (Wilcoxon; low vs. medium: *P*-adj = 0.006, low vs. high: *P*-adj = 1.1e-6, medium vs. high: *P*-adj = 0.006) (Figure 4A), there is no difference in 3'UTR-AEI between the three sub-groups (Figure 4B). Consistently, a significant correlation between ISG38 score and 3'UTR-AEI was observed in the control group and in the COVID-19 low viral load sub-group, but not in the COVID-19 medium and high viral load sub-groups (Pearson; control: *R* = 0.46, *P* < 2.2e-16, COVID-19 low viral load: *R* = 0.38, *P* = 0.0043, COVID-19 medium viral load: *R* = –0.1, *P* = 0.31, COVID-19 high viral load: *R* = –0.014, *P* = 0.92) (Figure 5). Our findings indicate that while higher viral load induces ADAR1 and other IFN-related genes expression, it barely changes the interferon response and the editing index. A reasonable explanation is that the editing activity during the disease is so high that it is nearly saturated, and cannot increase further even if ADAR levels continue to rise. Furthermore, in line with previous findings (49), it has been observed that in addition to the increase in ADAR1 expression, there is upregulation of other enzymes that compete for dsRNA binding proteins, consequently inhibiting some of the ADAR1 activity.

### Enhanced RNA editing in coding sites

**Figure 6.**
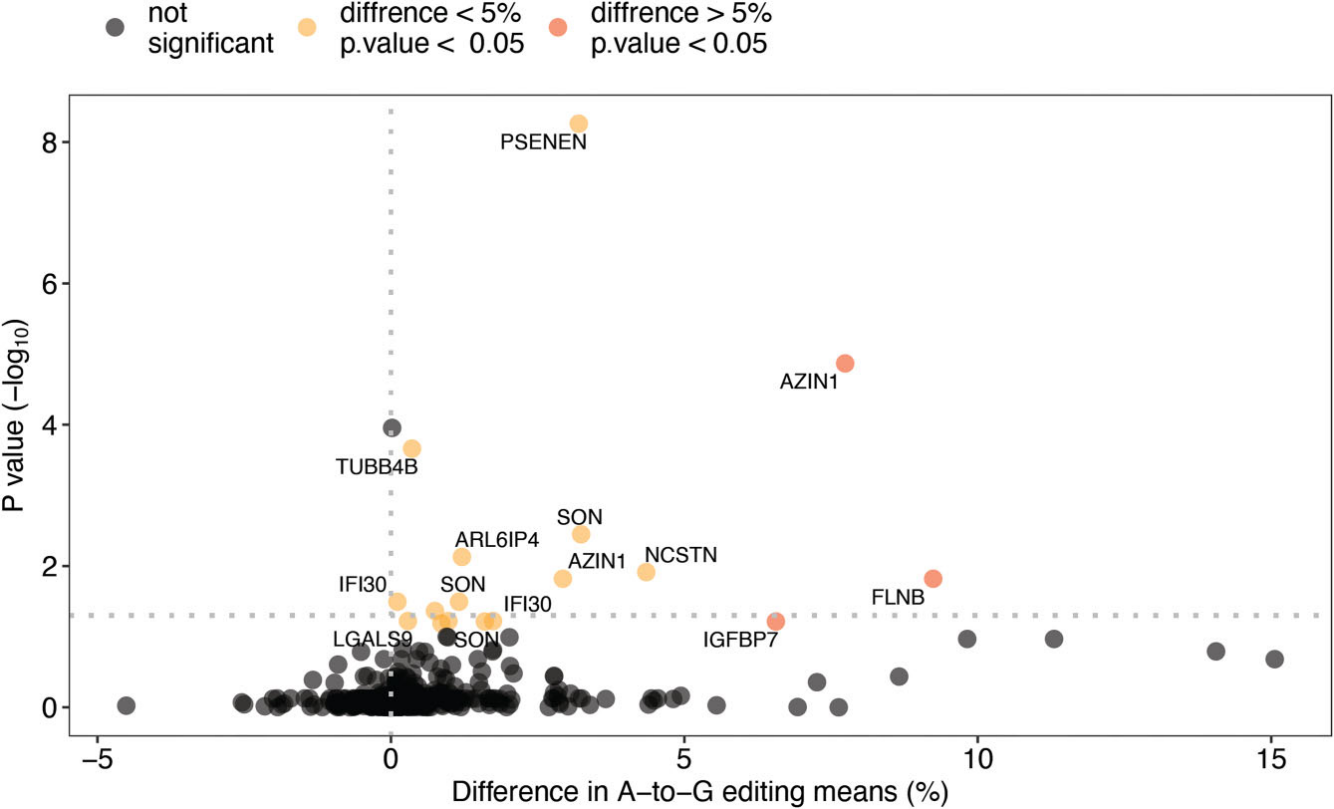
Editing differences between the COVID-19 group and the controls in 1517 coding sites that are prone to RNA editing. For each of the sites, the mean percentage of A-to-I editing was calculated in the COVID-19 group and in the controls. The difference in means between the two groups is depicted on the x axis (positive values indicate higher editing percentage in the COVID-19 group whereas negative values indicate higher editing percentage in the controls); sites of meaningful difference (defined as difference higher than 5%) are colored in red. The significance of the results is depicted on the y axis; sites of high significance (Wilcoxson *P*-value below 0.05) are colored in yellow or red. The 17 sites that exhibit statistically significant false discovery rate (FDR), and their corresponding gene symbols are labeled.

Although the majority of editing activity in mammals occurs in *Alu* elements, RNA editing is also found in coding regions. Such modifications are of high importance since they may potentially change the protein products. A list of 1517 coding sites that are prone to RNA editing was recently published by our group (12). Examining this list, we identified the specific sites in which editing differences between the COVID-19 group and the controls exist (Figure 6). In total, 17 sites of statistically significant editing difference between the groups were detected, located in 13 different genes. In all 17 sites, the editing levels was higher among the COVID-19 group compared with the control group. In 13 sites, the A-to-G substitution resulted in nonsynonymous or stoploss mutations (Table 1).

**Table 1.** Coding sites displaying notable editing differences between the COVID-19 group and the controls

Location	Gene	Strand	A-to-G SNV annotation	Editing percentage in the COVID-19 groupmean (median)	Editing percentage in the control groupmean (median)	*P*-value	FDR
chr1 160350197	NCSTN	+	nonsynonymous	0.085% (0.029%)	0.041% (0%)	0.00012	0.012
chr2 215376615	FN1	-	nonsynonymous	0.008% (0%)	0% (0%)	0.00074	0.043
chr3 58156064	FLNB	+	nonsynonymous	0.266% (0.246%)	0.174% (0.137%)	0.00018	0.015
chr4 57110068	IGFBP7	-	nonsynonymous	0.219% (0.189%)	0.153% (0.148%)	0.0015	0.061
chr8 102829408	AZIN1	-	nonsynonymous	0.204% (0.194%)	0.126% (0.103%)	3.90E-08	1.40E-05
chr8 102829409	AZIN1	-	synonymous	0.065% (0.035%)	0.036% (0.011%)	0.00019	0.015
chr9 137243170	TUBB4B	+	nonsynonymous	0.006% (0%)	0.002% (0%)	1.20E-06	0.0002
chr11 65583728	EHBP1L1	+	nonsynonymous	0.01% (0%)	0% (0%)	0.0011	0.06
chr12 122981654	ARL6IP4	+	nonsynonymous	0.021% (0%)	0.009% (0%)	6.40E-05	0.0074
chr17 27648981	LGALS9	+	stoploss	0.005% (0%)	0.002% (0%)	0.0013	0.06
chr17 38722707	MLLT6	+	nonsynonymous	0.013% (0%)	0.004% (0%)	0.0017	0.065
chr19 18173937	IFI30	+	synonymous	0.087% (0.07%)	0.071% (0.04%)	0.0013	0.06
chr19 18177741	IFI30	+	nonsynonymous	0.026% (0.013%)	0.025% (0%)	5.00E-04	0.032
chr19 35746748	PSENEN	+	nonsynonymous	0.044% (0.018%)	0.012% (0%)	7.80E-12	5.50E-09
chr21 33550530	SON	+	synonymous	0.014% (0%)	0.003% (0%)	0.00049	0.032
chr21 33550853	SON	+	nonsynonymous	0.033% (0.008%)	0.017% (0%)	0.0015	0.061
chr21 33551013	SON	+	synonymous	0.1% (0.095%)	0.068% (0.056%)	2.50E-05	0.0036

Interestingly, two of the sites are located on the IFI30 gene, which is directly linked to the Immune response Antigen presentation pathway and interferon gamma signaling (50,51). Another site is located on the PSENEN gene, which encodes the cellular protease gamma-secretase. Gamma-secretase was found to be affected by infection with Human Papillomavirus (52,53).

Though not completely understood at the moment, it is tempting to assume that even minimal editing in these detected coding sites is meaningful, and might have an impact by inducing a negative or positive feedback on these genes' expression or proper function.

### Global editing levels of post-COVID samples

**Figure 7.**
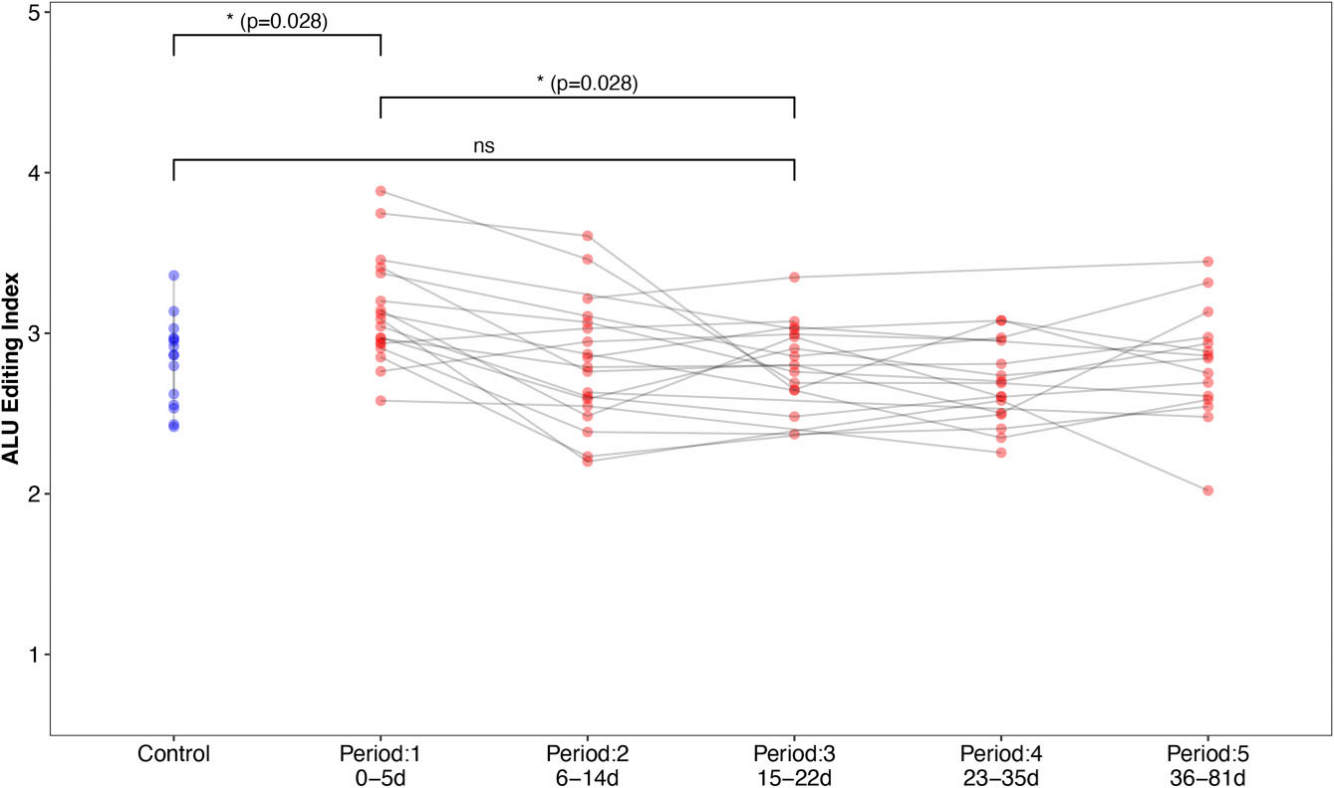
A-to-I editing in *Alu* elements in different periods post COVID-19. Calculated *Alu* editing index (AEI) in blood samples taken from COVID-19 patients in five different periods post diagnosis and from close-contact controls. Each grey line follows a COVID-19 patient. A significant AEI elevation is shown in the first period, but not in the third period, compared to controls.

To examine the long-term impact of COVID-19 on global RNA editing levels, we analysed an RNA sequencing dataset comprising blood samples from individuals infected with COVID-19 and non-infected close contacts. These samples were collected across five different periods following diagnosis. We found that the AEI was significantly elevated during the first period of the disease (0–5 days) compared to controls, and returned to baseline by the third period examined (15–22 days) (Wilcoxon; period-1 vs. controls: *P*-adj = 0.028, period-1 vs. period-3: *P*-adj = 0.028, period-3 vs. controls: *P*-adj = ns) (Figure 7). Our results indicate that the increase in global editing levels observed in the infected host is transient, and lasts about 3 weeks.

## Discussion

We showed a strong correlation between COVID-19 disease and elevated A-to-I RNA editing activity in the host.

To date, many viruses were reported to interact with the host's *ADAR* enzymes during the infection and induce RNA editing of the virus' genome (26,27). In this manner viruses can theoretically accumulate anti-viral or pro-viral mutations and obtain an evolutionary advantage. It was also reported that by activating host *ADAR* enzymes, viruses can also cause modifications in the host's RNA sequence as well (30).

To the best of our knowledge, Crooke *et al.* were the first to investigate A-to-I RNA editing levels in SARS-CoV-2 infected individuals and surprisingly reported reduced *Alu* dsRNA editing levels in severe COVID-19 patients (54,55). In the current work we analyze one of the same GEO datasets (GSE157103) and found opposite results. This unsuitability can be explained by the different criteria and techniques we used; Crooke *et al.* defined the proportion of A-to-I edits to exceed 5% of total reads at individual nucleotide site. However, as the typical per-site editing level is low (<1%), most sites are not identified using this coverage cutoff. Rather, summing the signal over all adenosines within *Alu* repeats results in a high SNR (ratio of editing events to mismatches of other sources) as we demonstrated recently (46) hence many more editing sites are included in the analysis.

Another research was performed by Light and Haas *et al.* (56), who reported significant upregulation of all *ADAR1* isoforms in SARS-CoV-2 human adenocarcinomic lung epithelial and alveolar basal epithelial infected cells. Using their experimental tool for detecting A-to-I RNA editing, they were able to show that non-repetitive hyper editing sites were much more abundant in SARS-CoV-2 infected cells compared to mock.

In the current research we examined RNA sequencing samples extracted from infected individuals, both of tissues infected by the virus directly (upper-respiratory tract samples) and indirectly (blood samples). We showed that though to a lesser extent, editing activity elevation was detected in the distant cells as well, indicating the substantial effect of COVID-19 infection on host RNA.

Viral-induced RNA modifications can theoretically cause severe outcomes. For instance, if the RNA sequence is being changed in coding sites or regulatory areas. This might be the key to better understanding the pathogenesis of post-viral infections adverse outcomes, such as autoimmunity, malignancy, or unexplained neurological manifestations as can sometimes be seen in COVID-19 (57). In our analysis, we detect higher RNA editing levels among COVID-19 patients in several protein-coding genes; One of them is even directly related to immune regulation and signaling as mentioned above (50,51).

We further show that the RNA editing activity elevation during the disease is temporary, as the levels return to baseline days/weeks post-infection. However, we hypothesize that even though the RNA sequence change is transient, it can still cause magnificent modifications to coding areas and result in long-term outcomes.

A thorough understanding of the immune system response to severe COVID-19 is crucial for developing effective treatments and novel therapeutic strategies in the effort to eradicate this fatal spreading pandemic. Here, we show that the ISG-38 score and *ADAR1* expression levels are elevated during the disease, even compared to other viral respiratory infections. In their article Yin *et al.* (58) show that the IFN response upon SARS-CoV-2 is primarily regulated by MDA5. *As* ADAR1 regularly interferes with the ability of MDA5 to bind long dsRNA molecules, it is reasonable to assume that MDA5 activation and IFN overexpression upon SARS-CoV-2, result in ADAR1 overexpression as a negative feedback regulator. ADAR1 overexpression, in turn, results in enhanced global RNA editing levels. Our results indicate that this pathway is enhanced during COVID-19 infection compared to other viral infections. Considering that the other viral infections encompassed several common cold coronavirus strains and influenza, it is conceivable that the results could vary slightly for each specific virus strain. Nevertheless, our findings affirm the concept that the immune response against SARS-CoV-2 is indeed highly robust.

In conclusion, COVID-19-infected individuals exhibit higher global A-to-I RNA editing levels than controls and other viral infections. These infection-induced epigenetic changes assumably contribute to the complexity of the immune system response and regulation during the disease, and might be related to adverse outcomes seen in post-viral cases, thus warrant further research.

## Supplementary Material

lqad092_Supplemental_File

## Data Availability

The data underlying this article are available in the database of Genotypes and Phenotypes (dbGAP), and can be accessed with accession #38851 and ID phs002258.v1.p1, and in the database of Gene Expression Omnibus (GEO) and can be accessed with accession #GSE166530, #GSE167000, #GSE171110, #GSE157103, #GSE166190. The source code used to produce the results and analyses presented in this manuscript are available on a GitHub repository at: https://github.com/zbidav/COVID19-rawData.git (https://doi.org/10.6084/m9.figshare.24164169.v1).

## Notes

Present address: Erez Y. Levanon, Mina and Everard Goodman Faculty of Life Sciences, Bar-Ilan University, Ramat Gan, Israel.
