# Supplementary material for: Elevated A-to-I RNA editing in COVID-19 infected individuals
Source: NAR Genom Bioinform. 2023 Oct 18;5(4):lqad092. doi: 10.1093/nargab/lqad092 (PMC10583280; doi:10.1093/nargab/lqad092)
Supplement: lqad092_Supplemental_File [file lqad092_supplemental_file.docx]

**Figure S1. *Alu* editing index and different baseline characteristics.**
A-B. Comparison between genders; No differences in *Alu* editing index (AEI) were detected between males and females in the COVID-19 group or in the control group, as well as in both groups combined.
C. No differences in *Alu* elements that are located in 3'UTR regions (3'UTR-AEI) were detected between males and females in the COVID-19 group or in the control group
D. Correlation between age and AEI; No correlation was detected, in both groups.


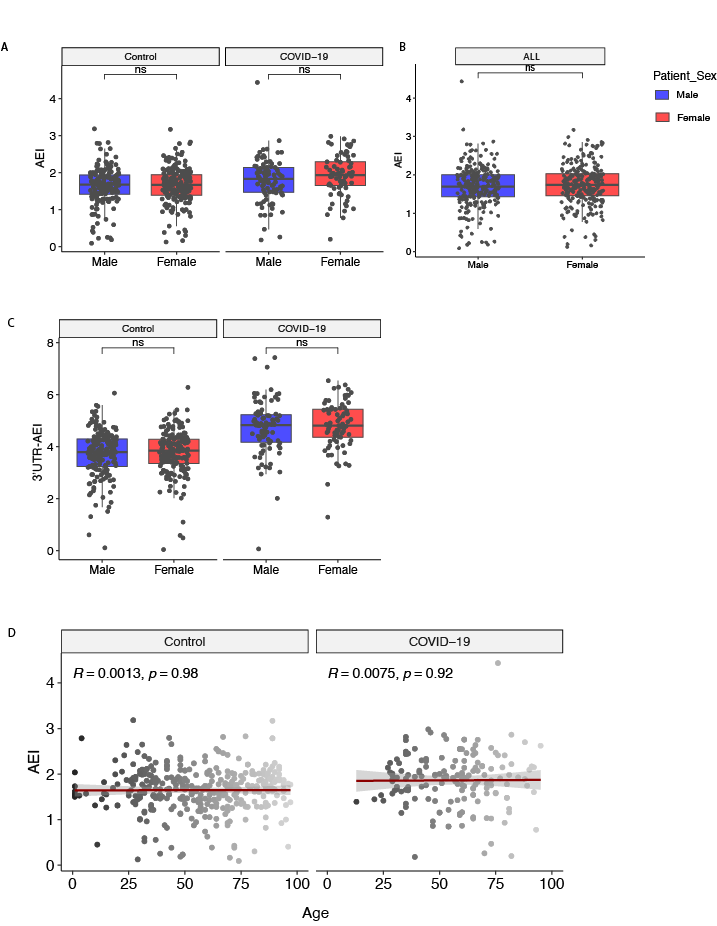


**Figure S2. Global A-to-I editing in *Alu* elements.**The *Alu* editing index (AEI) in nasal swabs of COVID-19 patients is significantly elevated compared to controls but not compared to the other-viral respiratory group.


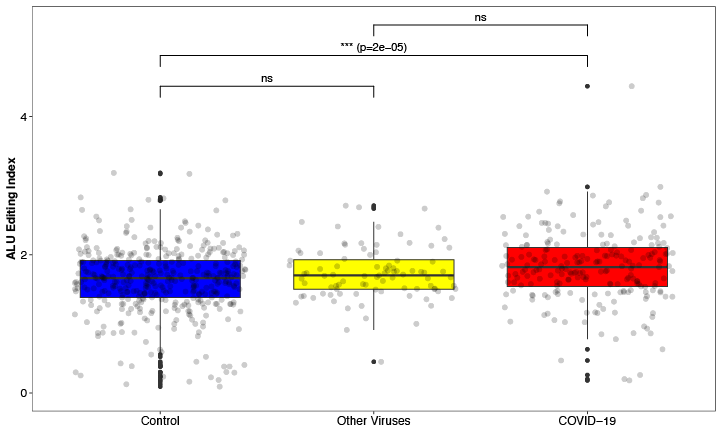


**Figure S3. ADAR1 expression levels.**
The expression levels of ADAR1 are shown for each of the small datasets separately. In all, ADAR1 expression levels are significantly higher in the COVID-19 group compared to controls.

**
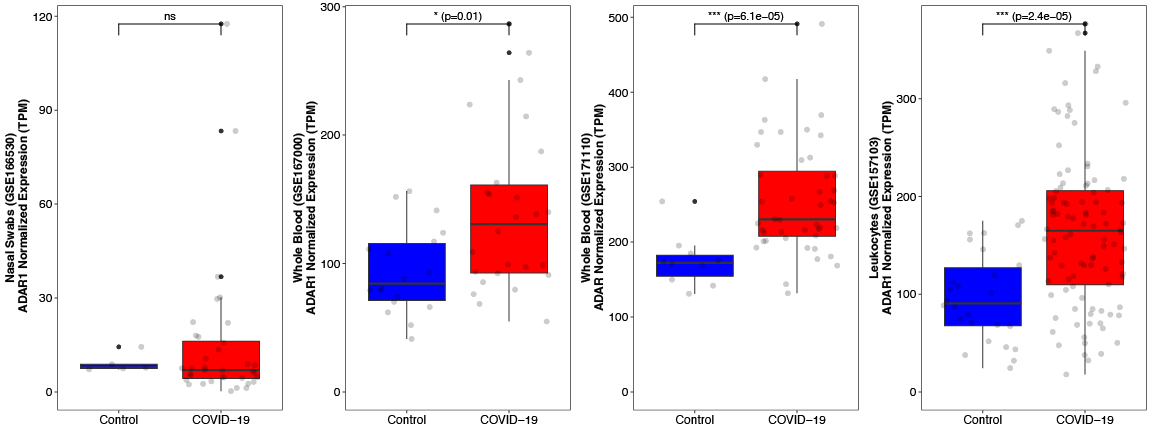
**

**Figure S4. Interferon Lambda and Beta gene expression levels.**
The expression levels of IFNL1 and IFNB are shown for the COVID-19 group, the other viral-respiratory group and the controls.

**
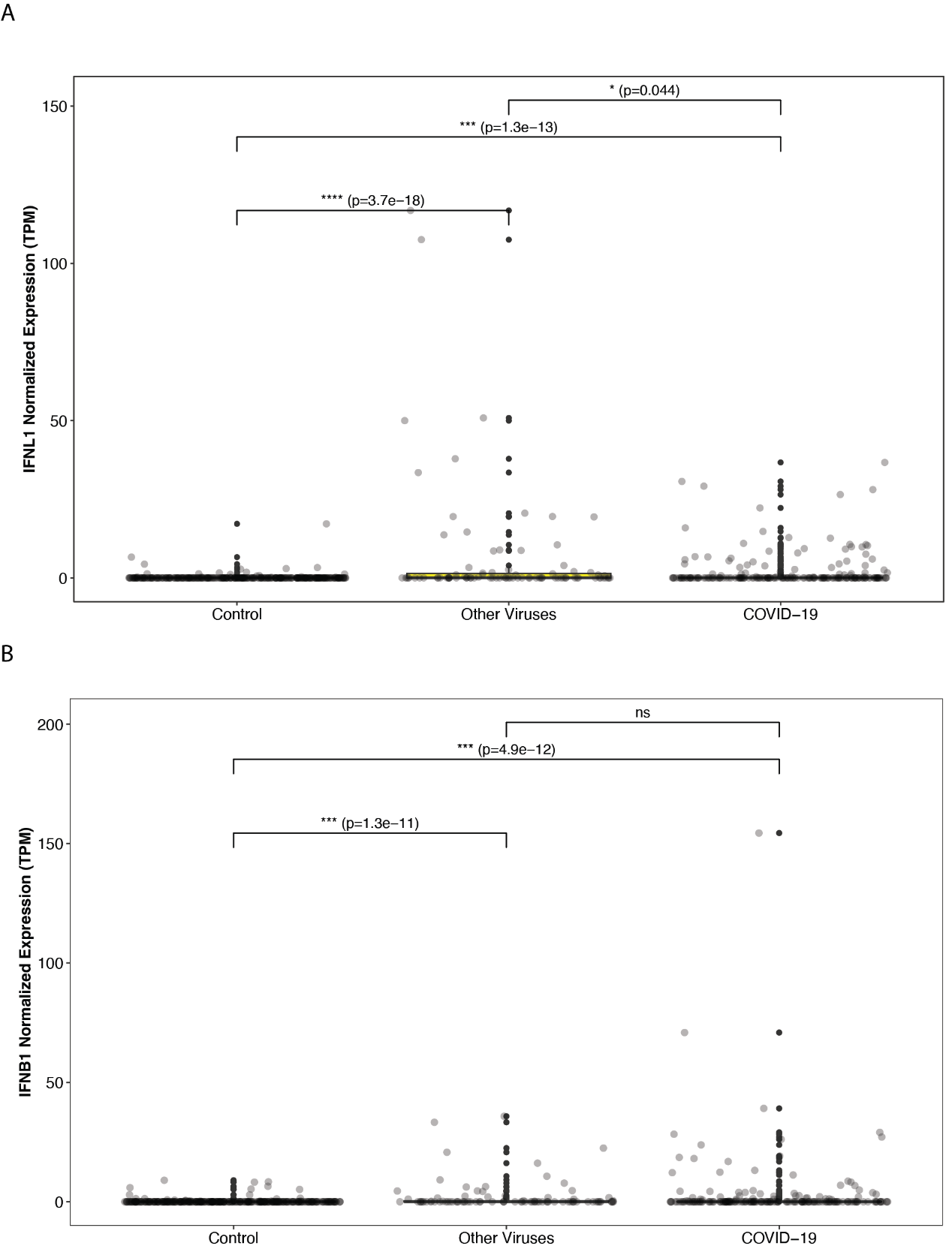
**
